# Sustainable and circularity in the decentralized hybrid solar-bioenergy system

**DOI:** 10.1007/s10668-023-03322-w

**Published:** 2023-05-11

**Authors:** Anthony Njuguna Matheri, Esther Nabadda, Belaid Mohamed

**Affiliations:** 1 Department of Chemical Engineering, University of Johannesburg, Johannesburg, South Africa; 2 Instituto de Bioingenieria, Universidad Miguel Hernandez de Elche, Elche, Spain

**Keywords:** Bioenergy, Bio-digester, Biomass, Decentralized, Integration, Solar energy

## Abstract

The sustainable development goals (SDGs) are fundamental to circular economy, climate action, sustainable digital environment initiatives that addresses the higher need of shifting towards fully sustainable and renewable energy systems. The decentralized power system has been applied and interconnected to the system and accounting to the renewable energy mix, energy storage and distribution. A decentralized hybrid renewable energy system can be of much help in providing a deficit of power between energy generation and demand where a single renewable energy system is not sustainable and reliable. The aim of this study was to characterize the biomass, perform the biomethane potential test of the biomass, select suitable bio-digester using multi-criteria decision analysis, carry out simulation-modelling of the solar-anaerobic digestion system in achieving energy self-efficiency with the development of the dynamic models that of climatic condition, social-economic and technology feasible. The performance of the system was designed and optimised to get maximum output power and mitigation of the environment at a lower cost. The performance was analysed based on present cost, locally availability, environmentally friendly, cost of energy on the local tariffs, load satisfaction, fuel consumption savings, operation and maintenance cost, human and technology capital and pollutants saving rates. The results showed major energy consummation from the solar-biodigester system, technology solutions that maximize energy efficiency and reduce the heat loss in the AD process. Waste to energy (WtE) technology coupled with the solar system could be viewed as key to sustainable, affordable and clean electricity generation.

## Introduction

**Fig. 1:**
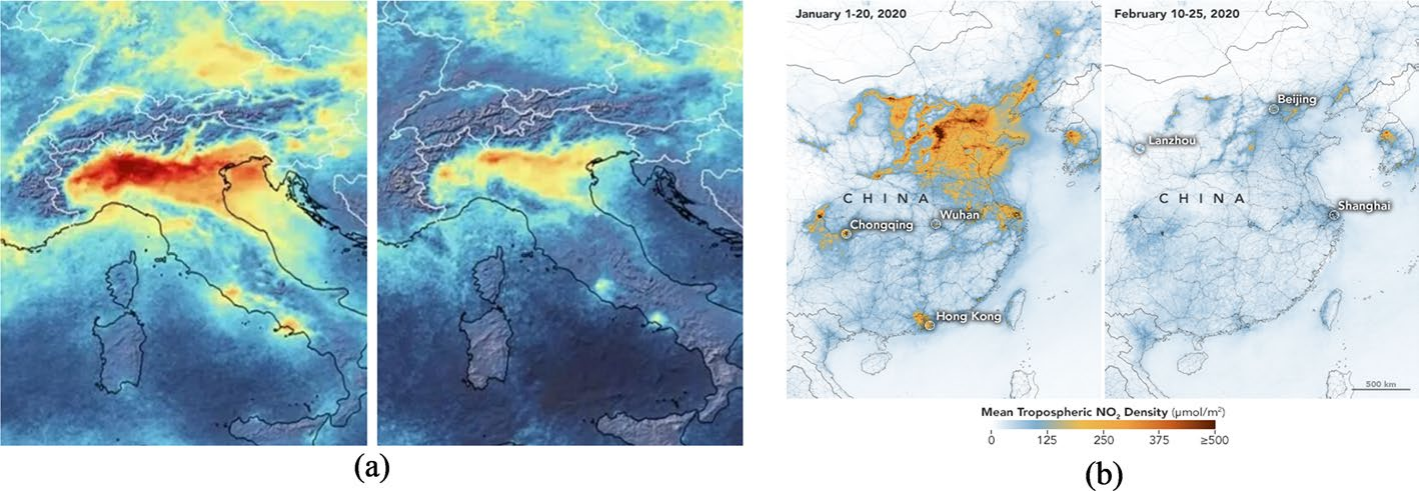
Satellite Image of the massive decline in pollution over **a** Italy and **b** China over the amid Corona Virus outbreak and quarantine (NASA Earth Observatory)

The energy consumption globally is experiencing a drastic increase in developing and developed countries due to industrialization and technological development with population growth. The disruption in the industry 4.0 is heavily dependent on the energy through the conventional sources that are not renewable and the emissions that have direct negative impacts on the climate change and environment through the greenhouse gases (GHG), carbon, nitrogen oxides, sulphur dioxide and unburnt hydrocarbon emissions. These gases contribute to global warming by increasing the temperature to above 3–6 °C of the pre-industrial levels (Anthony Njuguna Matheri et al., 2018; Njuguna Matheri et al., 2018; Rezzouk & Mellit, 2015). The United Nation Climate Change Conference (COP25), sustainable development goals-SDGs (ensuring reliable, affordable and sustainable energy for the people) and COP26 are the fundamental climate action initiatives addressing the need to move towards fully renewable, sustainable and low-cost energy system. Air, ice, land and water pollution are seeing a higher reduction across the globe and particularly China’s Industrial heartlands and Europe with crackdown of the Coronavirus (COVID-19) outbreak. Natural wildlife is returning home and adoption of the sustainable business processes, fintech, blockchain, artificial intelligence, hybrid of working from home and office taking shape due to COVID-19 disruption. This is initiated with low number of planes, cars, heavy and lower commercial vehicle, the heavy polluting industries in operations due to government regulations to curb COVID-19 spreading and thus less pollution. COVID-19 cleanses, purify and transfigure us all (Mother Earth is healing). The decline in pollution is attributed by the economic slowdown and travel restriction. The other incline is due to reduced use of fossil fuels, reduction in tourism numbers and masses at the streets. Figure 1 shows the satellite image of the massive decline in pollution over (a) Italy and (b) China over the amid ‘Corona Virus’ outbreak and quarantine (NASA Earth Observatory).

The deployment of the disruptive renewable energy mix (wind, solar, fuel cells, hydrogen, bioenergy) decreases the greenhouse gases (GHG) emissions where the pollution of the water and air continues to grow. The global initiative discourages the use of fossil fuel and adaptation to the smart climate-resilient and fully sustainable energy systems (Aghahosseini et al., 2019). The projected increase of population is more challenging in developing countries with limited supplies and distribution that is affordable and reliable. Mostly, developing countries depends on the natural resources and traditional biomass fuel for cooking that have high GHG emission. Adaptations of the alternative energy sources (renewable energies—RE) are more promising and serve as a substitute to fossil fuel (Aghahosseini et al., 2019).

Africa may have abundant energy resources but the continent is the home to least connected to the grid (electrification). The Sub-Sahara sunshine is said to be having enough energy to power the whole of Europe 7000 times over. The solar panel seems to be the solution as the panels reflect less heat into space reducing regional climate change and enhance increase rainfall. The concentrated solar power is much efficient in a dry environment, hot places but the steam generator needs much water and thus the need to integrate the energy, food and water nexus. Improvement of solar technology will lead to cheaper energy production that is efficient and sustainable. Solar panels would give Africa a solar superpower and turning it to be the global energy hub that solves geopolitics and enhance the realization of the industry 4.0.

### Bioenergy-anaerobic digestion

Biomasses from natural resources such as charcoal, firewood and crops are still a source of energy in many emerging economies and developing countries. Modern technologies are heavy burden to natural resources and this overburden the environment that lead to the pollutions from the emission of the GHGs (Neto et al., 2010). The rapid increase in the waste generation demand for a waste management system that provides cheap and sustainable innovative treatment solutions and technologies. Reliable integrated waste management (IWM) database provides all-inclusive resources on the evaluation of the waste management. Key knowledge of the biomass source, composition and end-to-end use is of necessity in life cycle assessment (LCA) of the IWM. Sustainable environmental management has six concepts; *rethink-repair-refuse-reduce-reuse-recycle* that lead to circularity of the zero waste*.* The waste hierarchy is incorporated in the waste-policy where the reduction of waste, reuse, recycle of the material and recovery is implemented. This answer the holistic question of; *reduce:* can the use of material be reduced?, *refuse:* are there materials a designer would not use?, *repair:* can the product be repaired instead of thrown away?, *recycle:* can materials be used that are easy to recycle when the product is finished with?. Sustainability thrive the dimensions through transparency transaction and analytics applications for entire life cycle. Application of the intelligent enterprise and accurate measure of the data driven environmental, social and governance (ESG) analytics is the first step to reduction of the organization footprint.

**Fig. 2:**
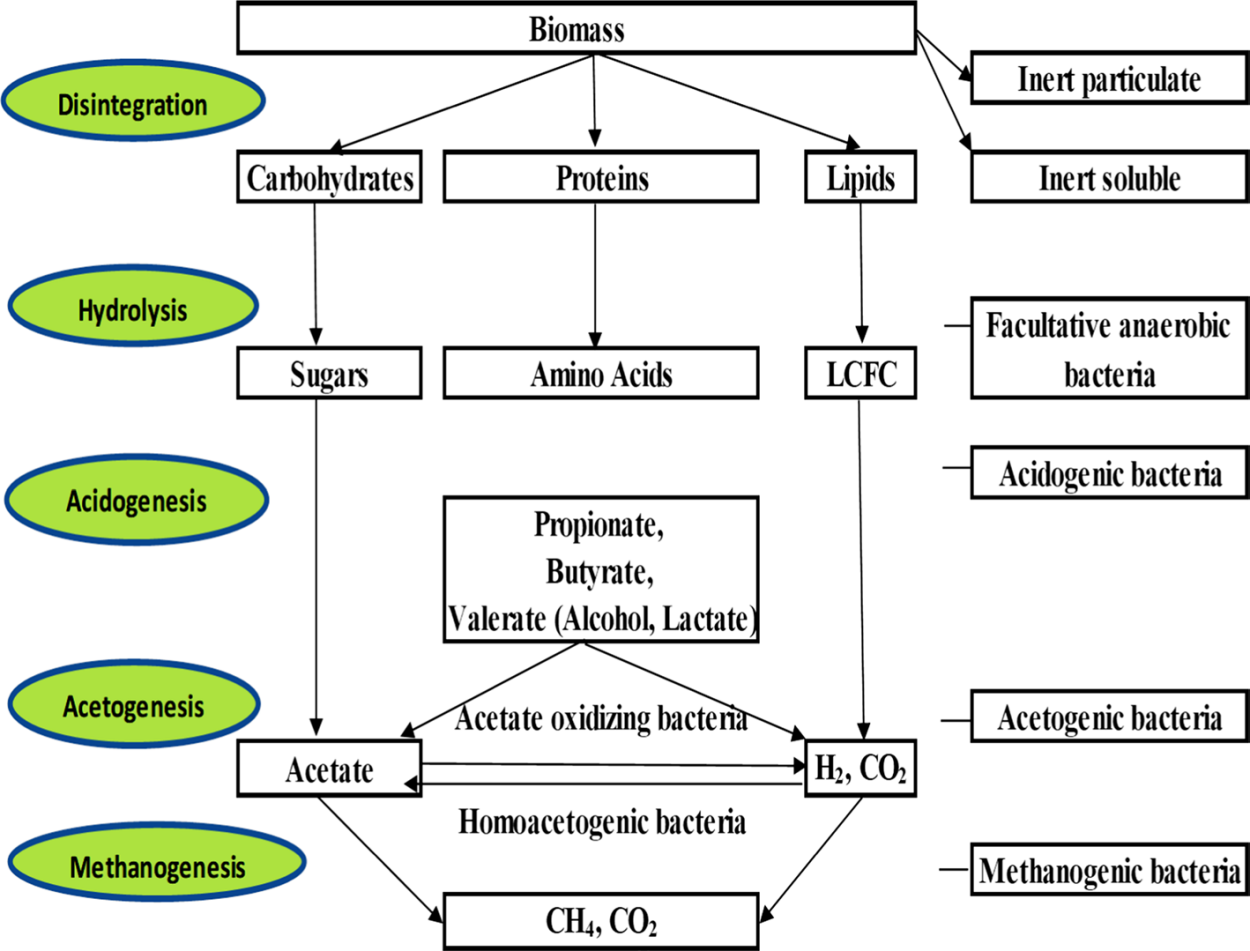
Degradation step of the anaerobic digestion process (Anthony Njuguna Matheri et al., 2016)

Adoption of waste to energy technology gives a pathway of an effective harnessing of the energy from biomasses and thus reduction of the GHG emission. The revised technologies include anaerobic digestion, fermentation, combustion, gasification, pyrolysis, liquefaction and incineration. Anaerobic digestion (AD) is a process where biogas/biomethane is formed by the break-down of organic fraction of the biomass using microbes. The composition of the biogas includes biomethane (60%), carbon dioxide (30%) with traces of the hydrogen sulphide, ammonia, carbon monoxide, oxygen and moisture content. Biogas can be stored in form of compressed natural gas (CNG) or liquid petroleum gas (LPG). A large number of companies are manufacturing biogas generators where others are for hybridization purposes. The parameters that affect the production of the biogas production include temperature, pH, organic loading rate (OLR), nature of nutrients, carbon–nitrogen (C/N) ratio, moisture content (MC), total solids (TS), volatile solids (VS), size of the digester, type of the digester, retention time, biomass pre-treatment, agitation, particle size, microbial balance, fatty acids, inhibitors (i.e. fatty acids, ammonia, toxic compounds) etc. Figure 2 shows the degradation stages of the *AD* process. The four pathways of the anaerobic digestion are; disintegration (inert particulate and soluble), hydrolysis (facultative anaerobic bacteria), acidogenesis (acidogenic bacteria), acetogenesis (acetogenic bacteria) and methanogenesis (methanogenic bacteria) (Anthony Matheri et al., 2017; Njuguna Matheri et al., 2018; Njuguna Matheri et al., 2018).

Bio-digestion is a clean and environmentally friendly innovative technology that will help improve the rural (remote) areas and informal settlements in the developing countries. Anaerobic digestion (AD) is sustainable, reduce organic waste disposal and generate RE. The digestate can be used as organic fertilizer that helps improve the soil nutrients and characteristics (Gaballah et al., 2020).

### Hybrid energy system (HES)

The decentralized energy system is suitable for the alternative power generation and is closer to the consumers. This overcome transmission losses that are inherently increased with the power transmission cables (PTC). When this power generation uses cheap, sustainable, renewable energies, there is a higher possibility to make locally potential resources that increase the rate of employment and income (Neto et al., 2010). Decentralized integrated or HRES can help provide a deficit of electricity between generation and demand for energy where single RE system is not reliable in the region. This will fulfils the COP25, proposed COP26 and United Nation sustainable development goals. The evolution of hybrid energy system (HES) consists of the power converter, photovoltaic (PV) modules, battery banks and a bio-digester. On the solar systems: PV modules is a direct current (DC) generator that consists of a variable number of photo-voltaic array cells connected electrically. The battery serves as a reservoir of the required load while the converter is the system used to convert AC power from DC. Among the hybrid system, the hybrid solar-bioenergy system is regarded as satisfactory based on economics, human capacity, locally available, technology transfer, thermodynamics and environmental evaluation. The local biomass and solar radiation resources utilization improve the usage of the renewable energy system and solving climate change mitigation. The life cycle assessment (LCA) is widely used to evaluate strategic planning, environmental impacts, eco-labelling programs and marketing. Life cycle cost analysis (LCCA) determine cost-effective option among different competing alternatives to own, purchase, maintain, operate, dispose and process. The life cycle energy assessment (LCEA) indicate that solar cells manufacturing requires more energy in generation than cost recovered by solar cells (Ali et al., 2019). The hybrid system (battery/PV/fuel cells/hydrogen/wind/grid/biogas) minimizes life-cycle cost, dump energy of remote areas and eliminate carbon emissions (Zhang et al., 2019). The concentrated solar power (CSP) in industry 4.0 is a proven technology system for energy generation at small, medium and large scale system (Khalid et al., 2017). This is enhanced by rethinking sustainable business process across circular value chain with sustainable intelligent enterprise that reduce carbon footprint.

To improve the quality of power and reliability of the systems, the energy storage, renewable energy technologies and backup systems are much needed. Stacking layers of the solar panels (compound II-V semi-conductor) increase energy efficiency by 50%. Control of air pollution (climate change) because of the restrict movement of people and vehicle have seen increase production of energy worldwide. RE resources exist on geographical areas that is wide. The rapid deployment of energy efficiency (EE) and RE results in climate change mitigation, energy security and economic benefits. Two or three power generation can increase sustainability, efficiency and reliability. Current commercially viable HRES include wind- diesel, PV- battery, PV- battery-wind, wind battery, PV- diesel, and lastly, the combination of PV- wind- diesel-battery systems (Bhandari et al., 2014). The higher the RE shares value in the market of the energy mix, the higher the flexibility needed for alternative back up for fossil fuel in ensuring power and heat availability on demanded (Blanchet et al., 2019).

A portable onsite waste solution (online waste solution) with an integrated system: the hybrid solar system and anaerobic digestion (AD) is a hybrid system that could reduce or eliminate electricity cost and even provide profit. This can serve as a solution to the integrated waste solution and power production in informal settlement, rural and remote location where getting reliable three-phase electricity supply is expensive and disruptive. Electricity production and distribution in developing countries usually come with higher flatulating tariffs with policies that changes over nights (political instability) together with lack of the intelligent network infrastructure.

**Fig. 3:**
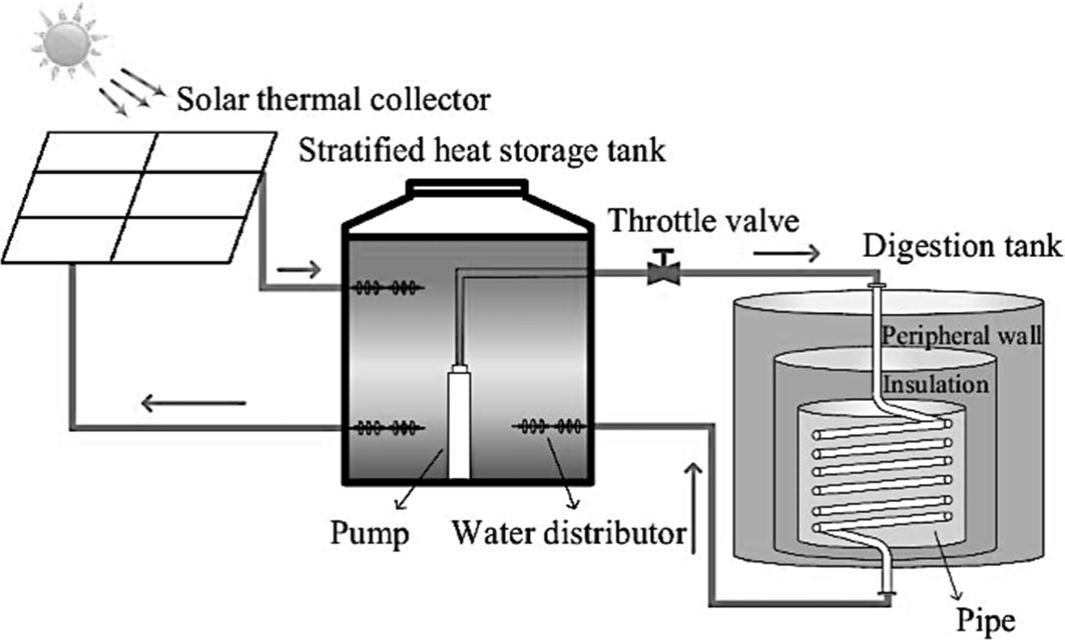
Schematic diagram of the integrated solar heating (thermal solar power) of the tubular anaerobic digester system (Chen et al., 2021)

Life cycle stages of PV involve the sourcing and production of raw material, manufacturing of modules, processing, balance of systems (BOS) components, installation of the system, decommissioning, the primary use of the systems, disposal and recycling (Ali et al., 2019). Figure 3 shows the schematic diagram of the integrated solar heating of the tubular anaerobic digester system (Chen et al., 2021; Gaballah et al., 2020).

PID of PV and biogas system model predictive controller (MPC) can be used to predict the behaviour of the dependent variables in the distributed power system (DPS) (Zhang et al., 2019). Individual low voltage on solar cell calls for the multiple cells to linked on the array (photovoltaics models or solar panels). The type and technical parameters of the measuring instruments are reviewed by (Kang et al., 2018). In the LCA of the HES, the policy-makers and stakeholders can obtain an understanding that is comprehensive to the system that is practically feasible and well-engineered (Zhang et al., 2019).

This study aimed to introduce a concept of solar-anaerobic digestion system in achieving energy self-efficiency with development of the dynamic models that of climatic condition, social-economic and technology feasible. The deployment of the system determined the greenhouse gases (GHG) emissions where the pollution of the water and air continues to grow. Key knowledge of the biomass source, biomass composition (characterization and quantification of biomass), biomethane potential test from different biomasses and end-use is determined and is of necessity in life cycle assessment (LCA) of the integrated waste management, sustainability, circularity and digitality of the ESG assessment and fulfilment of the SDGs. The type of the bio-digester selection was based on multi-criteria decision analysis (MCDA). The design of the solar-anaerobic digester was simulated. Simulation-modelling of the hybrid renewable energy system (energy mix) included solving the problem such as the efficiency of different systems, costing, sizing of the digester, availability of the biomass, energy input and output, optimization of the AD parameters and financial modelling. The rapid deployment of energy efficiency (EE) and RE will result to climate change mitigation, energy security and economic benefits that show visibility of the power generation with increased sustainability, efficiency and reliability The performance of the system was designed and optimised to get a maximum output power and mitigation of the environment at a lower cost. Life cycle cost analysis (LCCA) determined cost-effective option among different competing alternatives to own, purchase, maintain, operate, dispose and process. The revenue earned through the carbon credit in RE generation evaluate technology viability.

## Methodology

The section shows the characterization of the biomass, batch biomethane potential test of the biomass, use of the multi-criteria decision analysis to select suitable digester, simulation modelling of the bioenergy-solar-photovoltaic hybrid-system for the generation of the energy system, economic analysis of the system and CO_2_ emission analysis.

### Overall framework

**Fig. 4:**
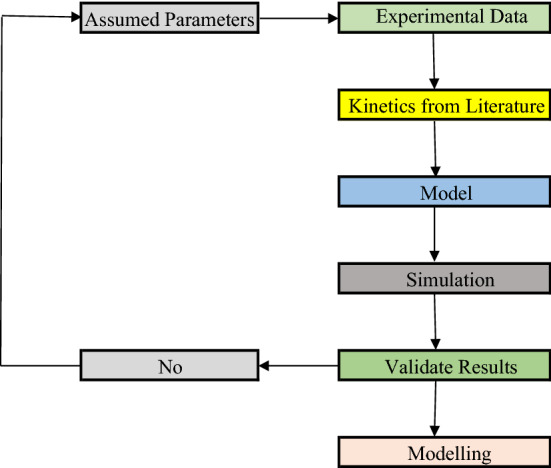
Dynamic modelling framework for sludge to energy

Figure 4 shows a framework of anaerobic digestion modelling and simulation.

**Fig. 5:**
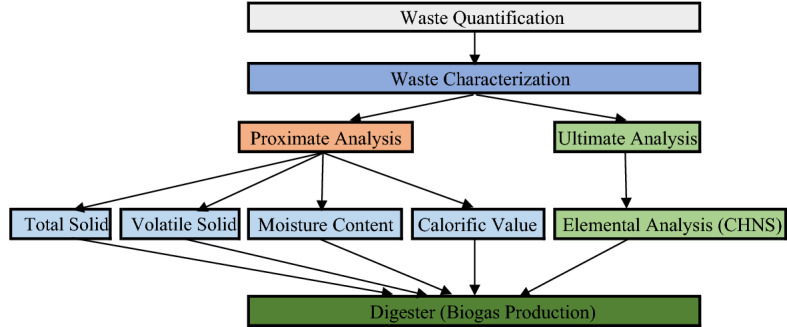
Flowchart for the biogas production

The flowchart model describes the methodology used in carrying out the waste quantification, characterisation and the analysis of the waste to energy *(anaerobic digestion).* Waste to energy underwent a bio-chemical process *(biogas/biomethane production using bio-methane potential (BMP) test technique)*. Carbonaceous and lignocellulosic waste was quantified and characterized for composition and sustainability. This was in accordance with the ASTM D5231-92 (Matheri et al., 2020). Figure 5 shows the flow chart of the waste quantification, characterization and biogas production.

The characterization and quantification were carried out in accordance to the ASTM D3173, ASTM D3302, ASTM D3175, ASTM E711/D5865, ASTM E870/D3176 (Matheri et al., 2016, 2020). The characterization was based on proximate analysis (MC%, VS% and TS%) while the ultimate analysis consisted of the carbon, hydrogen, nitrogen and sulphur (CNHS).

**Fig. 6:**
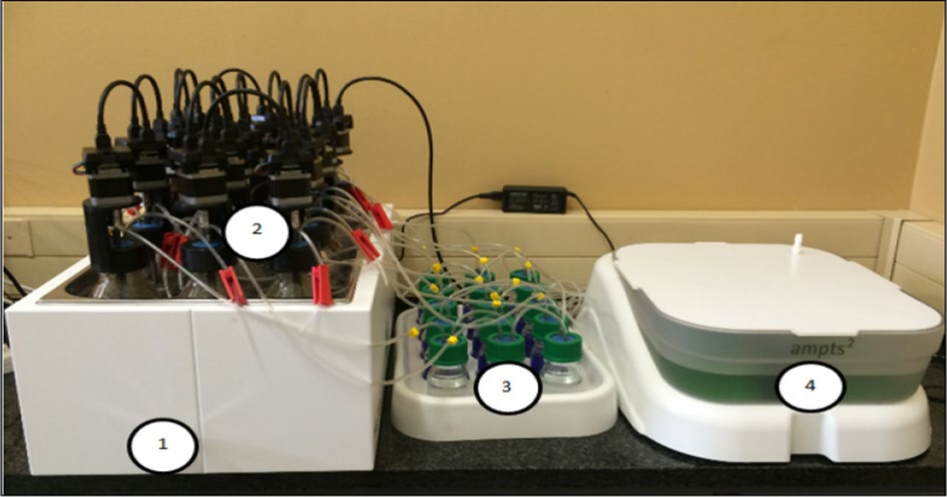
Biochemical methane potential set up with (1) water bath with thermo controller, (2) automatic bio-reactor (bio-digester), (3) CO_2_-fixing unit (scrubber unit), and (4) biomethane measuring system

Proximate analysis equation consisted of:

1 $$\mathrm{MC}\,\left(\%\right)=\frac{{M}_{\mathrm{wet}} -{M}_{\mathrm{dried}}}{{M}_{\mathrm{wet}}} \times 100$$

2 $$\mathrm{TS} \,\left(\%\right)=\frac{{M}_{\mathrm{dried}}}{{M}_{\mathrm{wet}}} \times 100$$

3 $$\mathrm{VS} \,\left(\%\right)=\frac{{M}_{\mathrm{dried}} -{M}_{\mathrm{burned}}}{{M}_{\mathrm{wet}}} \times 100$$

where *M*_wet_ was the sample mass before drying at 105 °C for 24 h with the oven, *M*_dried_ was the sample mass after drying at 105 °C for 24 h with the oven*, M*_burned_ was the sample mass after heating at 550 °C for 2 h with the furnace. Ultimate analysis equation consisted of:

4 $$\frac{C}{N}=\frac{\left(\mathrm{F }\times { C}_{f}\right)+(S \times { C}_{S})}{\left(\mathrm{F }\times { N}_{f}\right)+(S \times { N}_{S})}$$

where *F* was the first feed-stock, *S* was the second feed-stock, *C*_*f*_ was the carbon composition of the first feed-stock, *C*_*s*_ was the carbon composition of the second feed-stock, *N*_*f*_ was the nitrogen composition of the first feed-stock, *N*_*s*_ was the nitrogen composition of the second feed-stock. The biomethane potential (BMP) test was carried out using the bioprocess unit (automatic methane potential test system—AMPTS 11) and bioprocesses software. The methodology procedure was according to the AMPTS 11 bioprocess standard. Figure 6 shows the BMP set-up.

Pre-treatment of the biomass was carried into three phases; physical and mechanical through size reduction, thermal pre-treatment at 60 °C and biological pre-treatment by introduction of the inoculum. The organic loading rate of the biomass was determined based on the volatile solids. The samples were fed in the bio-digester in triplicate and pH adjusted using NaOH and H_2_SO_4_. The samples were flushed using nitrogen to make the system anaerobic. The digestion temperature was kept constant at 37 °C using a batch bio-reactor. The impurities were removed in the CO_2_ fixing unit using NaOH and indicator. Biomethane was collected at the measuring system using downward displacement method and recorded using Bioprocess automated and intelligent communication system. The triplicate results were reported on average.

### Design of the bio-reactor/bio-digester

The design of the digester was based on the organic loading rate (OLR) (Njuguna Matheri et al., 2018). Total weight of mixture:

5 $${W}_{t}=({W}_{\mathrm{vc}}+{W}_{\mathrm{vs}}+{W}_{\mathrm{vg}})+2$$

**Fig. 7:**
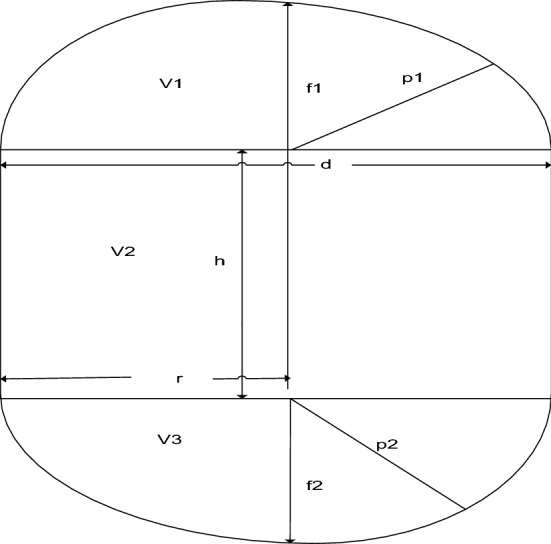
Bio-digester with gas and digestate collector domes

Figure 7 indicates the bio-digester with two domes.

The digester diameter was determined by:

6 $$d=(\sqrt[3]{{d}_{cap}}{)}^{1.173}$$

Digester volume of the top dome (gas collection unit):

7 $${V}_{1}=\frac{\left({3r}^{2}+{f}_{1}^{2}\right)*{\pi f}_{1}}{6}$$

where *V*_*1*_ was the digester gas collection unit (top part), *r* was the bio-digester radius and *f* was the height of the top dome. The digester volume:

8 $${V}_{2}=\pi {r}^{2}h$$

where *V*_*2*_ was the digester volume, *r* was the bio-digester radius and *f* was the height of the bio-digester.

Bio-digester volume of the digestate (bottom dome):

9 $${V}_{3}=\frac{\left({3r}^{2}+{f}_{2}^{2}\right)*{\pi f}_{2}}{6}$$

where *V*_3_ was the digester volume (bottom dome), *r* was bio-digester radius and *f* was the height of the dome.

The bio-digester surface area on the gas collection unit (top dome):

10 $${S}_{1}=2\pi {p}_{1}{f}_{1}$$

where *S*_1_ = was the bio-digester surface area (top dome), *p* was the bio-digester radius at the dome and *f* was the height of the dome. The digester surface area (digestion unit):

11 $${S}_{2}=\pi dh$$

where *S*_2_ was the bio-digester surface area (digestion unit), *d* was the bio-digester diameter and* h* was the height of bio-digester. The bio-digester surface area of the digestate unit (bottom dome):

12 $${S}_{3}=2\pi {p}_{2}{f}_{2}$$

where *S*_3_ was the bio-digester surface area of the digestate unit (bottom dome),* p* was the radius of the bio-digester and *f* was the height of the dome. The health, safety and environment (HSE) in operation were determined using the two surface areas of the cylinder and bottom sphere as:

13 $${S}_{a}=\pi d(0.5d+h)$$

The pressure was calculated as:

14 $$P=\frac{F}{\pi d(0.5d+h)}$$

The safety measure of the plant due to the pressure or stress was determined. It must be less than the bearing capacity divided by a safety factor and multiplied by the concrete strength as:

15 $$P<\frac{{b}_{cap}*{f}_{c}}{n}$$

where *n* was the safety factor of 10%,* f*_*c*_ was the concrete strength and *b*_cap_ was the bearing capacity. Equation 16 shows the expression:

16 $$\frac{F}{\pi d(0.5d+h)}<\frac{{b}_{cap}*{f}_{c}}{n}$$

### Solar system (photovoltaic (PV) solar)

Solar power comprised of the photovoltaic (PV) solar panels that collected energy and converted into DC electricity. The entire set-up of the solar panels included batteries, hybrid solar inverters, solar panels and mounting structure. Solar panels had anti-reflective, had a high conversion efficiency, anti-soiling surface that prevented power loss due to dirt and dust with excellent mechanical loading resistance. Hybrid solar systems consisted of both grid inverter and off-grid inverter system. A battery bank (power bank) was used. Structure monitoring accessories systems are much required with accessories (PV cables, AC cables, AC breaker, DC switches, AC/DC combine box).

The solar panels were the power sources for photovoltaic (PV) installation. The battery system was used for storing output power. A charger controller prevented over-charging and protected against over-voltage, which reduced the lifespan and performance of the battery. An inverter converted DC power to AC. It had an inbuilt protection against short circuit, overheating. To operate electrical loading, the DC power was converted into AC power. The battery storage bank was designed to feed loading up to days without the sunlight or dependant on the biogas with the system requirement. The inverter has inbuilt protection against low battery voltage, short circuit and, over-heating and over-load. Eight (8) different modules were selected by the energy management system (EMS).

### Simulation-modelling of the hybrid renewable energy system (Sustainable digital environment-SDE)

**Fig. 8:**
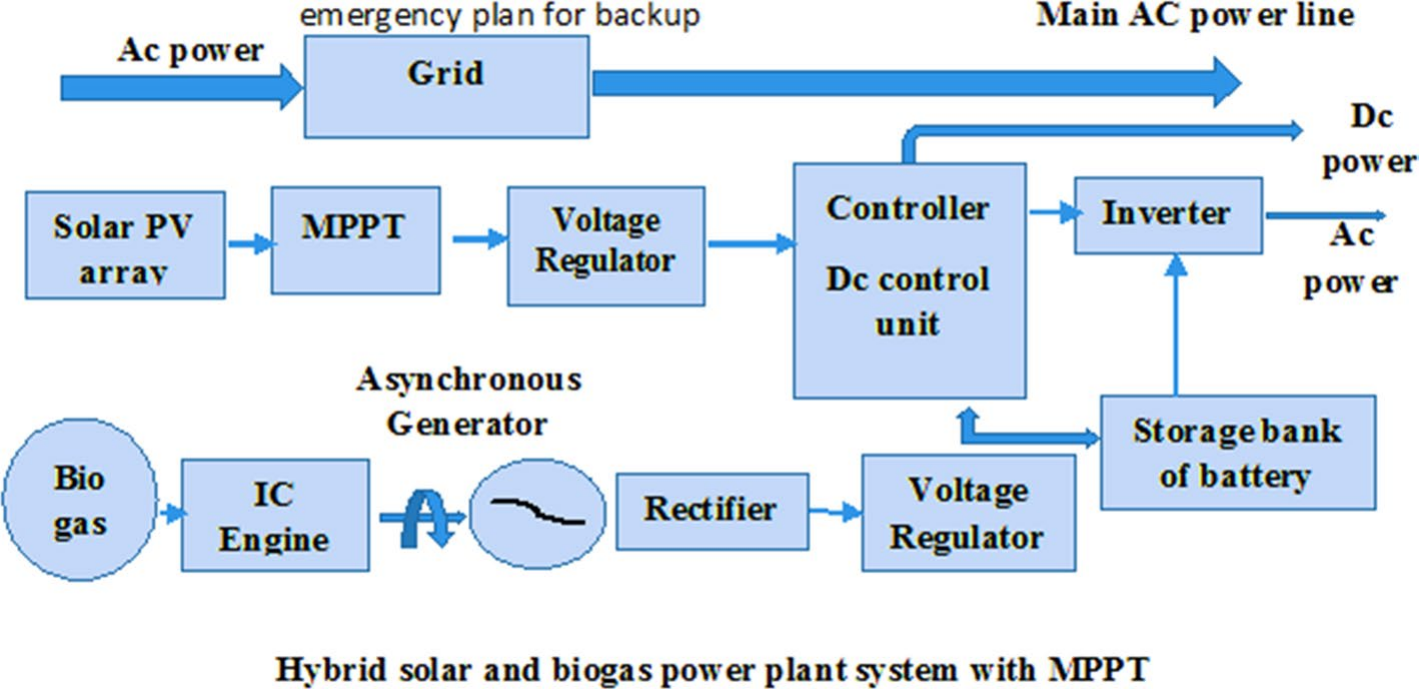
Schematic of the hybrid solar-biogas system (Rozario et al., 2015)

The hybrid system consisted of the two-system source of energy *i.e.* biomass and solar system to provide the system efficiency and balance in energy supply. Figure 8 shows the hybrid of solar-biogas system (Rozario et al., 2015).

Simulation-modelling of the hybrid renewable energy system (energy mix) included solving the problem such as the efficiency of different systems, costing, sizing of the digester, availability of the biomass, energy input and output, optimization of the AD parameters and financial modelling. The simulation was done with HOMER (hybrid optimization models electric renewable) energy, Microsoft Excel, MATLAB Simulink (programmable logic controller) software and model predictive controller (MPC).

#### PV panels

The maximum power point tracker (MPPT) was used with solar battery chargers to maximise power from the solar panel unit. The output power of the PV cells as a function of the temperate is shown in Eq. 17. Rate capacity of the solar panel arrays are calculated by (Rohani & Nour, 2014):

17 $${P}_{\mathrm{PV}}={Y}_{\mathrm{PV}}{f}_{\mathrm{PV}}\left[\frac{{G}_{T}}{{G}_{T,\mathrm{ STC}}}\right]\left[1+{\propto }_{P}({T}_{C}-{T}_{\mathrm{C},\mathrm{STC}})\right]$$

where *P*_PV_ was the array of the PV*, Y*_pv_ was the capacity rate of the PV array, *G*_*T*_ was the incident of the solar radiation in current condition, f_*PV*_ was the PV derating factor, *G*_*T*,STC_ was the radiation incident at standard test condition, *α*_*P*_ was the coefficient temperature of the power (%/°C), *T*_c_ was the temperature of the PV cell in the current condition (°C), T_*C*,STC_ was the temperature of the PV cell under standard test condition of the 25 °C. The MPPT not only maximised the system but minimised the return period of the total installation cost. The voltage power and current relation are nonlinear MPP. They are tracked for efficient extraction of solar energy in the PV system where individual tracking should be implemented.

#### Solar energy

Solar generated power is calculated by (Hassanalieragh et al., 2016; Zhang et al., 2011):

18 $${P}_{\mathrm{solar}}=A*{N}_{P}*{\eta }_{\mathrm{panel}}*\mathrm{Daily\,peak\,hours}$$

Or

19 $${P}_{\mathrm{solar}}={R}_{p}*{N}_{P}*\mathrm{Daily\,peak\,hours}$$

where the *P*_solar_ was the generated power by solar energy, *A* was the area of the solar panel, *N*_*p*_ was the solar panel efficiency (commercial efficiency 15%),* ɳ* was the number of PV panels and *R*_*p*_ was the rated power output of the selected panel and depends on the solar energy availability at a particular site.

Or

*E*, represents the PV array power generation (Kang et al., 2018).

20 $$E=\sum UIt$$

where *U* was the *PV* arrays output voltage (*V*), *t* was the time (s) and *I* is the array output current.

#### Energy storage-battery bank

The energy storage-battery bank (ESBB) with Total Nominal Capacity *(C*_*n*_*)* was permitted to discharge up to the limit defined by the Depth of Discharge (DOD%) at the maximum permissible limit (Upadhyay & Sharma, 2015).

21 $${C}_{\mathrm{min}}=DOD*{C}_{n}$$

The present state of a battery *(C*^*d*^*)* was dependant on the load power requirement and diesel generator energy production (Upadhyay & Sharma, 2015).

22 $${C}^{d}(t)={C}^{d}(t-1)+{n}_{B}\frac{{P}_{B}^{d}}{{V}_{BUS}}t$$

where *C*_*d*_ (*t*–1) was the capacity energy storage at time (*t*–1)th in hour, *t* was the simulation time step *i.e. t* = 1 h*, V*_bus_ was the bus voltage for the DC*,* n_B_ was the round trip efficiency of the battery with the charging capacity of 80% and 100% at discharge, *P*_B_^d^ was the power battery input/output (*W*) < 0 is the discharge and > 0 is the charging, and *T* was the time for the simulation.

#### Energy saving rate

The energy-saving rate due to renewable energy conserving measures (Kang et al., 2018).

23 $$\eta = \frac{{W_{1} - W_{2} }}{{W_{1} }}*100\%$$

where the *W*_*1*_ and *W*_*2*_ were the consumption of renewable energy before and after the adoption of the measures to energy-saving, respectively. This occurs by reducing coal consumption.

#### Economic analysis

Economic analysis was utilized with HOMER. Two parameters were put into account: net present cost (NPC) and the cost of electricity (COE). The NPC presented the cost of a system during the project lifeline *i.e.* 20 years. This included the cost of the operation and maintenance, fuel replacement, initial construction, salvage cost (remaining value of each component at the end of the project) and penalties due to emissions.

##### Net present cost

The *NPC* was presented by:

24 $$\mathrm{NPC}=\frac{{C}_{\mathrm{tot}}}{\mathrm{CRF}}$$

where NPC was net present cost, CRF was the capital recovery factor in ratio of the present value to an annuity and *C*_tot_ was the total annualized cost (TOC) which was the sum of the annualized costs of each system component (Khalid et al., 2017; Rohani & Nour, 2014).

25 $$CRF=\frac{[i({1+i)}^{n}]}{[(1+i{)}^{N}-1]}$$

where *i* was the annually real interest rates, 6% on current. Bigger NPC is caused by the interest rate drop that causes a reduction in the recovery of the initial capital. *N* was the number of 5 years.

##### Bank loan repayment

Annuity of the repayment of the loan is calculated by (Chong et al., 2011):

26 $$A=\frac{\mathrm{PV}*r}{[1-(1+r{)}^{-n}}$$

where *A* was the annual payment, PV was the present value, *n* was the number of period and *r* was the rate of interest.

##### Internal rate of return

Equation 27 shows the internal rate of return (IRR):

27 $${\text{NPV}} = \left( {\mathop \sum \limits_{t = 1}^{T} \frac{{C_{t} }}{{(1 + r)^{t} }}} \right) - C_{o}$$

where NPV was the net present value, *C*_*o*_ was the cost of the total initial investment, *C*_*t*_ was the net cash in-flow during the period *t, r* was the *IRR* and t was the period time (Chong et al., 2011). The *IRR* is presented as:

28 $$\mathrm{IRR}=\frac{(\mathrm{Cash\,flows})}{(1+r{)}^{i}}-\mathrm{initial\,investment}$$

where *IRR* was the internal rate of return, cash flow in the period, *I* was the period time and *r* is the discount rate.

##### Cost of electricity

Equation 29 shows the calculation of the cost of electricity

29 $$\mathrm{COE}=\frac{{C}_{\mathrm{tot}}}{E}$$

where *COE* was the cost of electricity, *E* was the efficiency, *C*_tot_ was the total annualized cost (TAC) (sum of the annualized costing of each component in the system) (Khalid et al., 2017).

#### Emission of CO_2_

*CO*_*2*_ emission from the generator is an important criterion in the design. It is calculated by (Upadhyay & Sharma, 2015):

30 $${E}_{t}=\sum_{t\varepsilon T}\sum_{n\varepsilon N}{E}_{n}{gn}_{nt}$$

where *E*_*n*_ was the amount of generated CO_2_ emission by the unit* n* in time *t* in kg/kWh and *gn*_*nt*_ was the cumulative energy output of non-renewable generating n unit in time t in kWh.

## Results and discussions

### Quantification and characterization of biomass

**Fig. 9:**
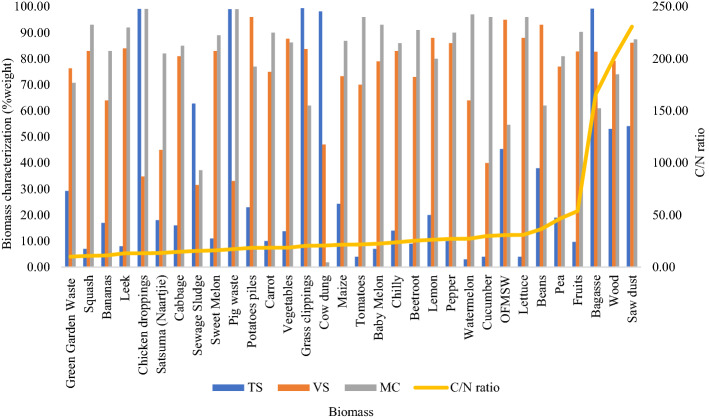
Proximate and ultimate characterization of the substrates

The biomass samples were analysed for the proximate and ultimate. The results indicated the properties and characteristics of the feedstock that were of the optimum range and promising for the waste to energy conversion (Fig. 9). High TS%, MC% and VS% showed that the feedstocks are easily biodegradable. The amount of moisture content ranging from 60 to 90% influence the AD process (biomass degradability) and biomethane formation. High dilution of the feedstock had a negative impact of the bio-digester design and AD processes. The wetter the substrate, the higher the bio-digester volume and area that took relatively huge level of the produced biomethane. The total solids of the biomass were within the required range as this influenced the performance of the anaerobic digestion and changed the microbial morphology in the digestion system. The use of the bulk substrate reduced the digestion volume as observed by (Anthony Njuguna Matheri et al., 2021). High volatile solid of the substrates gave an indication of high conversion of substrate to biomethane. The biodegradability indicated that the biomass was feasible for the anaerobic digestion (biogas/biomethane production). The C/N ratio from CHNS was an important factor in food (nutrients)-microbial balance of the AD process.

Most substrates had a C/N ratio that was on the optimal range of 15–30, except for the bagasse, sawdust and wood with C/N ratio of 165.81, 230.89, 200.64, respectively*.* Optimum C/N ratio indicated a nutrient balance required to conduct the anaerobic digestion. Animal manure, grass clippings and agriculture waste indicated a good potential for the biomethane production. Higher C/N ratio in substrate required co-digestion with the lower C/N ratio that was below the optimum range. A comprehensive feedstock quantification and characterization was important. This helps in finding optimum conditions or parameters for optimum anaerobic digestion and biogas/biomethane production and in designing of the biodigesters.

### Mono and co-anaerobic digestion

**Fig. 10:**
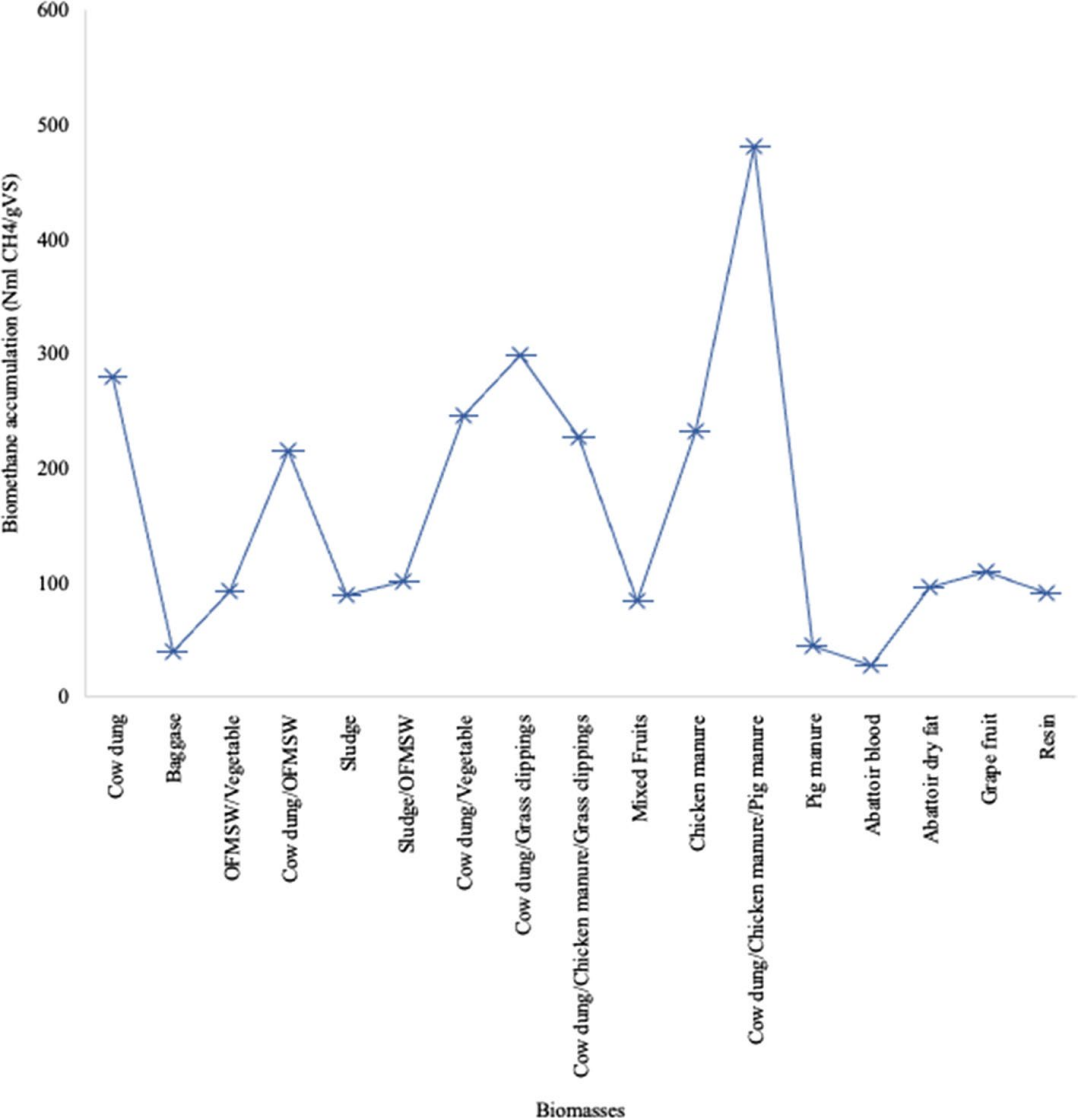
Biomethane production per volatile solids

Biomethane potential test was determined using AMPTS 11 with different biomass as feedstock (organic fraction of the municipal solid waste (OFMSW), animal waste, agriculture waste, sewage sludge) and recorded in NmlCH4/gVS. The average of the triplicate results was recorded for the batch biomethane potential test. Lower production on lag phase was due to the lower solubility of the substrate and enzymes during the anaerobic digestion processes. High production of biomethane was recorded after 5 days of hydraulic retention time (HRT). The temperature was kept constant at the mesophilic temperature of 37 °C, constant agitation, optimum pH and feeding rate calculated according to the volatile solids of the substrates. Figure 10 shows the biomethane production per volatile solids of the mono and co-digestion substrates.

Lower production of the biomethane was recorded with a substrate that had lower or above range (15–30) C/N ratios. Higher C/N ratio created nutrients imbalance to the microbial due to lower nitrogen of the substrate thus lowering the pH down. High C/N ratio lower biomethane production due to a formation of carboxylic acids that inhibited microbial growth and shift micro-organism environment. Low C/N ratio leads to lower biomethane production due to production of ammonia (high nitrogen in the substrate) with high pH that poison the digestion process. Lower volatile solids substrates recorded lower production because of lower nutrient. Higher volatile solids recorded higher production of the biomethane. Co-digestion of the substates improves the production because of balance of the nutrients, pH optimization, food to microbial balance, regulated C/N ratios, balance VS, TS and MC as observed by (Singh et al., 2018). This was observed with the highest production of biomethane with the co-digestion of cow dung, chicken manure and pig manure. Bagasse waste produced lowest biomethane due to difficulty of biomass degradability and higher C/N ratio. Co-digestion reduced the HRT with an increased production rate. Mono digestion was only possible with substrates that had an optimum condition from characterization. Co-digestion was highly recommended to balance the nutrients optimally high level of control. Optimum and constant mesophilic temperature ensured a controlled atmosphere for the digestion.

### Selection and design of bio-digester

**Fig. 11:**
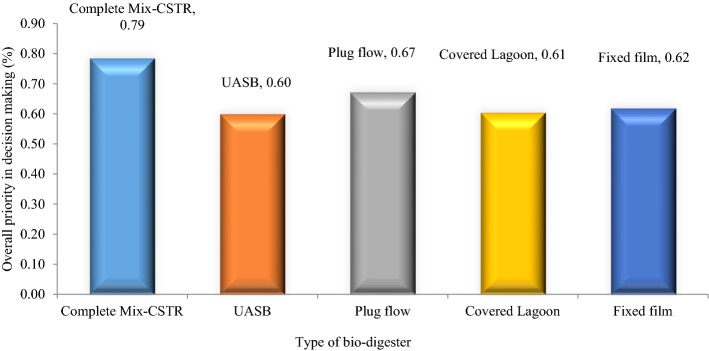
Selection of the bio-digesters using multi-criteria decision analyser technology

The type of the bio-digester was selected by use of multi-criteria decision analysis (MCDA). This was based on the presence of the agitation accessory, local availability, substrate suitability, scalability, ease of construction and maintenance and temperature regulation. Figure 11 shows the selection of the bio-digesters using multi-criteria decision analyser technology.

The UASB, coverage lagoon, fixed film, and plug flow scored 60%, 61%, 62%, 67% respectively with the complete mixed continuous stirred tank reactor *(biodigester-CSTR)* scoring highest with 79%. The CSTR fixed doomed was selected for the production of the biomethane. Table 1 shows the bio-digester merits and demerits with the installation cost of CSTR fixed dome, CSTR floating dome and plastic CSTR.

**Table 1:** Bio-digester merits and demerits with the installation cost

Digester	Fixed dome digester	Plastic digester	Floating dome with plug flow
Merits and demerits	Life span of 20 years	Biogas production during maintenance	Easy to clean
	High level of experience	Easy to partly replace	Biogas production during maintenance
	Big range of total solids	No agitation	No agitation
	Difficult to clean	Life span of 15 years	Life span of 15 years
	No biogas production during maintenance	Difficult to clean	High total solid
	Gas leaks	Container size limitations	Leak sensitives
Cost	R250000	R150000	R137500

Floating dome digester recommended more advantages than the fixed dome and plastic digester. The merits were on the high bio-digester lifespan, high range of total solids, easy to clean, ease of maintenance during digestion operation, lesser cost on agitation and embedded with high leak sensitivity.

### Solar panel-mesophilic digestion

The mesophilic digestion temperature was at a constant rate in running the bio-digester. The variation was simulated throughout the year on two seasons. Winter recorded lower temperature because of frequently cold frost and thus required activation energy for the digestion from an external source). Summer day required lesser energy to heat the processes (Ouhammou et al., 2019). The excess generated energy on winter time could be used directly in the house *i.e.* cooking, lighting, drying or water treatment. The bio-digester was equipped to run at mesophilic temperature of the 37 °C. Auxiliary energy (thermostat) was needed to heat and compensate heat loss in winter while summer time showed excellent solar efficiency and performance. The solar-AD system design covered the heating requirement of the AD processes that required ambient temperature with the prevention of the convection and radiative heat transfer losses.

**Fig. 12:**
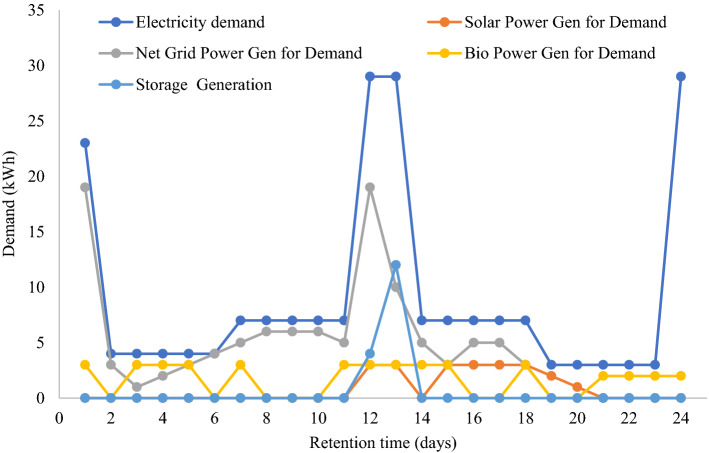
Supply and demand per energy source of the hybrid AD-PV solution

The PV parameters included the area of panels, area of the roof, peak power, the variation of the solar variation and capacity power while the AD parameters included the proximate and ultimate parameters. Other parameters were bio-power generation demand, solar-power generation demand, electricity demand, storage energy demands and grids power demand. Figure 12 shows the supply and demand per energy source of the hybrid AD-PV solution. The installation of the storage capacity of 30 kW, bioenergy of 2.5 kW with no CHP and solar energy (15 panels) of 5 k with approximately R250000 as initial cost.

From the observation, biogas production increased by volume with increased solar energy generation. The increase of the solar energy increase the optimal required temperature for the mesophilic digestion. There was high electricity demand with lower net grid power generation for the demand recorded on 1–2, 11–13 and 23–24 day respectively due to energy dissipation. The PV power generation system achieved a conversion efficiency of 8.1%. The energy savings improved from 60 to 90%. The process saved R5000 per month with 3 years energy of payback time (EPT). The installation system reduced CO_2_ emission by approximately 25 t. (Su et al., 2017) reported that a proper selection of the ratio of carbon/steam to the optimal direct CO_2_ footprint increases the system exergy efficiency. The model can generate optimal cost solution that meets the demand with optimum tariffs, intelligent network infrastructure and advanced policies coupled with political stability. Waste to energy (WtE*)* technology coupled with the solar system could be viewed as key to sustainable, affordable and clean electricity generation, circular bioeconomy, water-energy-food nexus, liquid biofuel for the transport sector and industrial, climate change mitigation and adaptation, food security (digestate use as organic fertilizer), independent power producer (IPP) programme and more to conservation of the environment with social and governance integration.

### Cost for solar panel installation

In avoiding frequently load shedding (power rationing) and avoiding dependence or reliance on the national power grids), disturbance of change of weather (natural parameters), hefty investment is required into solar power systems and bioenergy (waste management, water utilization and fertilizer production-food security). Solar power comprised of the photovoltaic (PV) solar panels that collected energy and converted it into DC electricity. The entire set up of the solar panels included batteries, hybrid solar inverters, solar panels and mounting structure. An 80 m^2^ house was estimated to require 2 kW of power per day. The cost was $4500 (14 Rands to 1 Dollar). The solar power systems based on monthly electricity bills in South Africa were summarized as shown in Table 2.

**Table 2:** Solar power systems based on monthly electricity bills in South Africa

Electricity expenditure per month	System size	Approximate price
Below R1300	2 kW	$4500
Between R1300 and R2200	3 kW	$5285
Between R2200 and R5000	5 kW	$7857
Over R5000	10 kW	$13,428

The economic feasibility and installation of the systems and breakeven were dependent on individual power usage. Combining AC inverter (*e.g.* Tesla Powerwall) makes a continuous power supply. Successful implementation of the system can open avenues for the replicates to other developing countries and emerging economies thus saving valuables, enhancing integrated waste management, producing fertilizers (reduce starvation and food security) and electrification. This will kick-start a virtual cycle of economic growth by providing the most pivotal and empowering services related to social-economic impacts. The decentralized energy system on renewable energy gave a significant contribution to ESG in an informal settlement and rural areas by replacing traditional form of energy to the hybrid renewable energy system (HRES). The HRES independently provide a power source that is stable from solar and biogas energy.

### Computational for emission mitigation

Equivalent certified emission reduction (CER) of 1 ton of CO_2_ as computational with the amount of mitigation of equivalent carbon mission per day/year with the hybrid power plant was observed. The revenue earned through the carbon credit in RE generation made the technology more viable.

## Conclusions and recommendations

Thermal performance of the solar-anaerobic digestion was explored. Temperature fluctuations with energy input were observed to be having greater input on the unit system. The results prove high efficiency of current energy systems and feasibility of the mesophilic digestion of the waste to energy technology (waste benefaction). The technology was uncomplicated and durable. The hybrid power plant could assist in load shedding/power rationing season. Bio-digester provided cleaner and more affordable sources of energy to traditional or underserviced community. The installation was of the storage capacity of 30 kW, bioenergy of 2.5 kW and solar energy (15 panels) of 5 k with approximately R250000 as initial cost. Biogas production increased by volume because of constant temperature. The PV power generation system achieved a conversion efficiency of 8.1%. The energy savings improved from 60 to 90%. The process saved R5000 per month with energy payback period (EPP) of 3 years. The installation reduced CO_2_ emission by 25 t. The models generated an optimal cost solution that met the demand with optimum tariffs, intelligent network infrastructure and advanced policies coupled with political stability. The HRES is relatively a cost-effective solution in areas where the utility grids are expensive and thus suitable for the informal settlements and remote (rural) regions. Integrating distributed generating system in a mini-grid increased the efficient distribution of power and reliability. The proposed hybrid system could be considered as clean (green) development and disruptive innovative technology with adequate technical support and funding.
